# Two-Dimensional Polarized Blue P/SiS Heterostructures as Promising Photocatalysts for Water Splitting

**DOI:** 10.3390/molecules29184355

**Published:** 2024-09-13

**Authors:** Yin Liu, Di Gu, Xiaoma Tao, Yifang Ouyang, Chunyan Duan, Guangxing Liang

**Affiliations:** 1 Department of Physics, School of Science, Guangdong University of Petrochemical Technology, Maoming 525000, Guangdong, China; liu.yin@gdupt.edu.cn; 2 School of New Energy and Environmental Protection Engineering, Foshan Polytechnic, Foshan 528137, China; 3 School of Physical Science and Technology, Guangxi University, Nanning 530004, China; taoxiaoma@gxu.edu.cn (X.T.); ouyangyf@gxu.edu.cn (Y.O.); 4 Shenzhen Key Laboratory of Advanced Thin Films and Applications, Key Laboratory of Optoelectronic Devices and Systems of Ministry of Education and Guangdong Province, State Key Laboratory of Radio Frequency Heterogeneous Integration, College of Physics and Optoelectronic Engineering, Shenzhen University, Shenzhen 518060, China; lgx@szu.edu.cn

**Keywords:** blue P/SiS heterostructures, internal electric field, photocatalytic water splitting, first-principle calculations

## Abstract

Two-dimensional (2D) polarized heterostructures with internal electric fields are potential photocatalysts for high catalytic performance. The Blue P/SiS van der Waals heterostructures were formed from monolayer Blue P and polar monolayer SiS with different stacking interfaces, including Si-P and P-S interfaces. The structural, electronic, optical and photocatalytic properties of the Blue P/SiS heterostructures were studied via first-principle calculations. The results showed that the Si-P-2 or P-S-4 stacking order contributes to the most stable heterostructure with the Si-P or P-S interface. The direction of the internal electric field is from the $\left(001\right)$ surface toward the $\left(00\bar{1}\right)$ surface, which is helpful for separating photo-generated electron–hole pairs. The bandgap and electrostatic potential differences in the Si-P-2(P-S-4) heterostructures are 1.74 eV (2.30 eV) and 0.287 eV (0.181 eV), respectively. Moreover, the Si-P-2(P-S-4) heterostructures possess suitable band alignment and wide ultraviolet and visible light spectrum regions. All results suggest that 2D polarized Blue P/SiS heterostructures are potential novel photocatalysts for water splitting under a wide ultraviolet and visible light spectrum region.

## 1. Introduction

Hydrogen production from the photoelectrochemical splitting of water in sunlight, producing clean and versatile hydrogen fuel along with oxygen, is considered to be a potential solution to the global energy crisis without environmental concerns [1,2,3]. There is neither a consumption of non-renewable fossil fuels, nor a production of harmful environmental by-products [4,5]. Since a method using a semiconductor electrode was developed for water photolysis in 1972 [6], the solar-driven photocatalytic water splitting technique has received extensive attention from researchers. The mechanism of photocatalytic hydrogen generation from water mainly involves three processes. Firstly, the catalyst absorbs a portion of sunlight and generates photogenerated charge carriers. In the solar spectrum, light with energy greater than the bandgap width is harvested, while that with energy lower than the bandgap width is not absorbed. Secondly, although most of the photogenerated charge carriers recombine and annihilate during migration to the catalytic surface, some of them migrate successfully. Thirdly, the photogenerated charge carriers that reach the catalyst surface undergo redox reactions with water, producing hydrogen and oxygen [7,8]. It is well known that effective solar light harvesting and carrier separation are essential for improving the efficiency of the photocatalytic water-splitting process. However, due to the traditional photocatalysts having narrow absorption spectrum ranges and low efficiency of charge carrier migration, the hydrogen production rate and solar-to-hydrogen (STH) conversion efficiency of photocatalytic water splitting are still less than expected. The hydrogen production rates of ZnS samples and ZnS/CuFeS_2_/ZnO films are 232.7 μmol [9] and 615 μmol [10], respectively. The solar-to-hydrogen (STH) conversion efficiency of SrTiO_3_:Al, SrTiO_3_:La,Rh/Au/BiVO_4_:Mo, and InGaN/GaN nanowires are 0.76% [2], 1.1% [11], and 6.2% [3], respectively. In the current experimental stage, this is still far from being ready for industrial application. The photocatalyst is critical for improving the efficiency of water photolysis. Previously, TiO_2_ and ZnO were applied as photocatalysts under ultraviolet (UV) light (wavelength of 100–400 nm) which only constitutes about 7% of sunlight [12]. Due to the wide bandgaps between the valence band maximum (VBM) and the conduction band minimum (CBM) in TiO_2_ [13,14,15] and ZnO [16,17], the inactivation of photocatalysis under visible light (wavelength of 380–700 nm) and infrared (IR) light (wavelength > 700 nm) limits their absorption efficiency in sunlight, which contains a large proportion of visible and infrared light. Visible light cannot be utilized by a photocatalyst unless its bandgap is narrower than 3 eV [18]. Developing photocatalytic materials with relatively narrower bandgaps to efficiently harvest irradiation with a broader range of wavelengths is demanded by the improvement of water splitting using solar energy.

The bandgap of a photocatalyst was considered to be larger than 1.23 eV for water photolysis [19], the variation value of redox potentials of water; however, infrared light cannot be absorbed within this range of bandgap. This problem can be solved by an internal electric field being introduced by the intrinsic dipole within a two-photocatalyst system [12,20,21]. The internal electric field separates the electron–hole pair and redox reactions to opposite surfaces of the system and shifts the CBM along with the reduction potential of water to be less positive on the electron-receiving surface to produce hydrogen [22,23,24,25,26]. Shifting the bands on the hydrogen-producing surface results in band bending between two surfaces. This band bending reduces the variation between CBM and VBM, and between the redox potentials of water. As a result, the bandgaps of this two-photocatalyst system are narrower than 1.23 eV. Yang’s study also conducted the first principle density functional computation and indicated that the surface-functionalized boron-nitride bilayer with a large intrinsic dipole may be a promising photocatalytic material for hydrogen production using infrared light [12].

The mechanism of an internal electric field in bandgap reduction has inspired many studies on 2D materials with polarity [27,28,29,30]. Monolayer Janus MoSSe, a transition metal dichalcogenide material with an asymmetric structure, has been synthesized and has shown activity of hydrogen production [31]. Besides experimental synthesis, asymmetric Janus MXY (M = Mo, W, Pt, or Si; X, Y = S, Se, or Te; X ≠ Y) 2D materials have been studied theoretically, indicating promising potential in water photolysis [32,33,34,35,36,37]. Other Janus materials, such as X_2_PAs (X = Si, Ge, or Sn) [38], Al_2_XYZ (X/Y/Z = S, Se, Te, X ≠ Y ≠ Z) [39], and HfSnX_3_ (X = S, Se or Te) [40], have also been reported to be good candidates for solar-driven water splitting. Group IV-VI monolayers [41,42,43,44,45], consisting of elements in these two groups (group IV: C, Si, Ge; group VI: O, S, Se, Te), have been studied for their electric structure and potential strong absorption in visible light [46,47,48,49]. Miao’s research group reported that 2D silicon chalcogenides, for example, the SiX (X = S, Se, Te) monolayers, exhibit promoted carrier mobility, which could improve the efficiency of photocatalytic hydrogen production from water [50]. Additionally, our previous study found that the 2D polar monolayer silicon monochalcogenide SiS involves a polarization electric field generated by the dipole moment [44]. This polarization electric field is able to separate photogenerated charge carriers quickly. Therefore, 2D polar monolayer silicon monochalcogenide SiS is considered to be a potential photocatalyst for water splitting. However, the polar monolayer SiS is an indirect semiconductor with a bandgap of 2.997 eV [44], which is relatively large, limiting the efficiency of solar absorption. To improve the efficiency of this material in water photolysis, the solar spectrum absorption range needs to be broadened, which could be achieved by constructing 2D heterojunctions. Building heterostructures is a useful way to improve the optical properties of photocatalysts [51,52,53,54,55,56,57]. Polar materials with heterostructures may show advantages introduced by polarity and heterostructure [58,59,60,61,62]. The photocatalytic performance of 2D polar monolayer silicon monochalcogenide SiS is enhanced by its high charger carrier mobility and polarization electric field. Additionally, blue phosphorus (Blue P) [63,64,65] displays a similar structure and lattice constant to that of monolayer SiS. Therefore, a heterojunction can be constructed between SiS and Blue P, resulting in the regulation of bandgap width and the expansion of the solar spectrum absorption range. Thus, the limitation of monolayer SiS is overcome. Therefore, in order to improve the efficiency of photocatalytic water splitting, forming heterostructures based on polarized Blue P/SiS would be a promising strategy, which could enhance solar light harvesting and improve carrier separation simultaneously. Polarized Blue P/SiS heterostructures exhibit both high charge carrier mobility and a broader solar spectrum absorption range, making them highly efficient photocatalytic materials. Thus, the heterostructures formed by SiS and Blue P are worthy of evaluation for their electrical and optical properties. 

## 2. Results and Discussion

**Figure 1:**
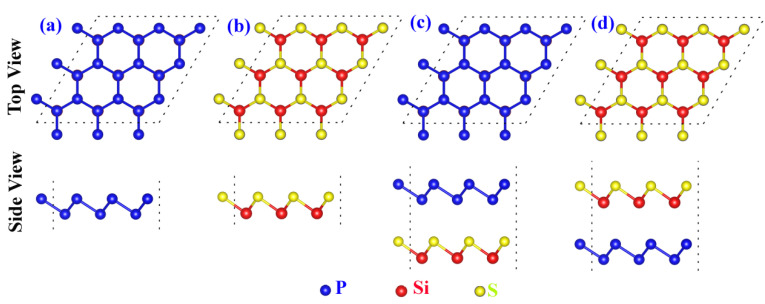
Top and side view of a crystal structure. (**a**) Monolayer Blue P; (**b**) Monolayer SiS; (**c**) Blue P/SiS van der Waals heterostructures with a P-S interface stacking order; (**d**) Blue P/SiS van der Waals heterostructures with a Si-P interface stacking order.

The atomic structures of monolayer Blue P (Figure 1a) and the monolayer SiS (Figure 1b) display similarity to graphene with a hexagonal honeycomb lattice. However, the side views of these two materials exhibit a zig-zag structure (Figure 1a,b), which differs from graphene. Structural parameters of monolayer Blue P and SiS calculated in this work are shown in Table 1. The lattice parameter (*a*) of monolayer Blue P and SiS are 3.27 Å and 3.30 Å, respectively, which agree with previous studies [50,66,67,68]. It is worth noting that the difference between the lattice constants of monolayer Blue P and SiS are very small. 

The heterojunction, the atomic interface between two distinct materials, can be formed vertically between monolayer Blue P and monolayer SiS via van der Waals interaction, as indicated by the variation between their lattice constants (*a*) which is only 0.03 Å (an about 0.9% mismatch). This means that monolayer Blue P and SiS are easily constructed into 2D van der Waals heterojunctions in the vertical direction. Heterojunction establishment by stacking Blue P and SiS vertically should consider the relative locations of P atoms to Si or S atoms in monolayer SiS. Different interfaces between distinct layers could impact the structure as well as the properties of the photocatalytic materials. Stacking Blue P on top of SiS (Blue P-SiS) would build the Blue P/SiS van der Waals heterostructures with a P-S interface (Figure 1c), while SiS/Blue P van der Waals heterostructures with a Si-P interface would be formed by positioning Blue P below SiS (Figure 1d). 

Twelve highly symmetrical stacking orders were evaluated in the current study (Figure S1), including six for the P-S interface (P-S-1, P-S-2, P-S-3, P-S-4, P-S-5, P-S-6), and six for the Si-P interface (Si-P-1, Si-P-2, Si-P-3, Si-P-4, Si-P-5, Si-P-6). As shown in Figure S1a,g, the Si atoms are located at the hexagonal center of the Blue P layer, while the S atoms are located on the top or bottom of the P atoms. As shown in Figure S1b,h, all the Si and S atoms are located on the top or bottom of P atoms, so the hexagonal honeycomb lattice of monolayer SiS coincides exactly with that of monolayer Blue P. As shown in Figure S1c,i, the S atoms are located at the hexagonal center of the Blue P layer, while the Si atoms are located on the top or bottom of the P atoms. As shown in Figure S1d–f,j–l, these monolayer SiS sequences in stacking orders 4 to 6 can be generated by rotating the monolayer SiS in stacking orders 1 to 3 (shown in Figure S1a–c,g–i) horizontally by 180°.

The stability of the heterostructure is affected by the stacking order. The energies of the Blue P/SiS van der Waals heterostructures built by various stacking orders after a full relaxation were compared to identify the most stable heterostructures and the corresponding stacking order. As shown in Figure S2a, stacking order 2 for the Si-P interface (Si-P-2) has the lowest energy value, suggesting that the Si-P-2 stacking order heterostructure is the most stable of the Si-P interface heterostructures. As shown in Figure S2b, the energy value of stacking order 4 for the P-S interface (P-S-4) is the minimum, suggesting that the P-S-4 stacking order heterostructure is the most stable of the P-S interface heterostructures. Therefore, the Si-P-2 and P-S-4 stacking heterostructure are further discussed in the rest of this article.

**Figure 2:**
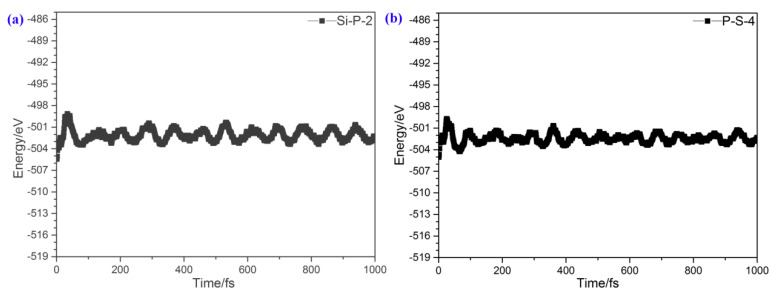
Time-dependent evolution of the total energy in AIMD simulations at 300 K for Blue P/SiS van der Waals heterostructures. (**a**) Si-P-2 and (**b**) P-S-4 stacking orders.

To study the stability of the 2D polarized Blue P/SiS heterostructure, the time-dependent evolution of the total energy was calculated using the AIMD simulations. The Blue P/SiS heterostructure with 5 × 5 × 1 supercells, which possess 100 atoms, was adopted in the simulation. The total simulation time was 1000 fs, with a time interval of 1 fs. As shown in Figure 2a,b, regardless of whether they are Si-P-2 or P-S-4 stacking orders, the total energy of the Blue P/SiS heterostructure fluctuates slightly between −501 and −504 eV, with a mean value of about −502.3 eV, suggesting the dynamic stability of Blue P/SiS van der Waals heterostructures.

**Figure 3:**
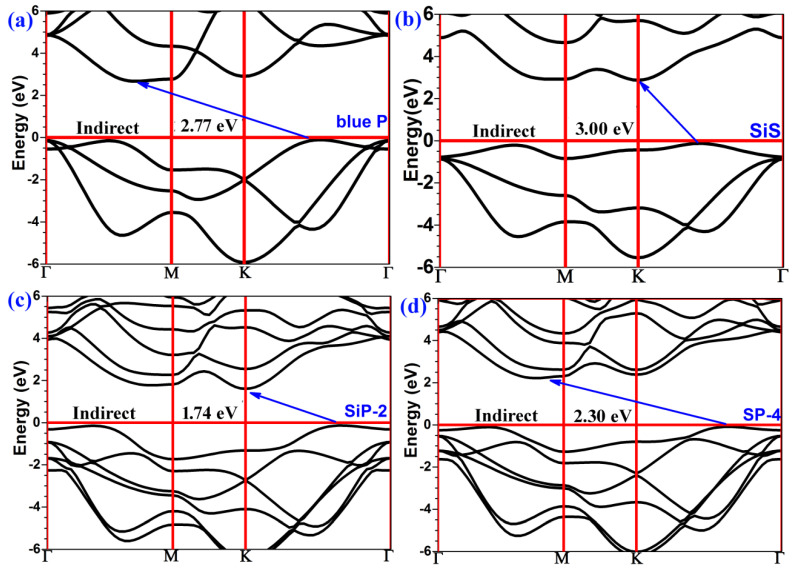
The electronic band structure of (**a**) monolayer Blue P; (**b**) monolayer SiS; (**c**) Blue P/SiS van der Waals heterostructures with the Si-P-2 stacking order; and (**d**) Blue P/SiS van der Waals heterostructures with the P-S-4 stacking order.

The electronic band structure is one of the most important properties of semiconductive materials. An appropriate bandgap is necessary for an ideal photocatalyst. Light utilization induced by a wide bandgap only occurs in a small range of UV light, limiting the photocatalytic efficiency. However, a narrow bandgap may not satisfy the energy band positions needed for producing hydrogen and oxygen through water splitting. The electronic band structure was computed using Perdew–Burke–Ernzerhof (PBE) functional and Heyd–Scuseria–Ernzerhof (HSE06) functional methods in this work (Table 1). Although the computed bandgap varied depending on the methods, the evolution trends in electronic band structure were consistent between the two methods. Unlike the PBE method which underestimates the bandgap, the HSE06 method adjusts the handling way to the Coulomb repulsion treatment, resulting in more accurate bandgap results. The band structures of monolayer Blue P, monolayer SiS, and the Blue P/SiS van der Waals heterostructures with Si-P-2 and P-S-4 stacking orders were calculated using the HSE06 method (Figure 3). 

As shown in Figure 3a, the monolayer Blue P has an indirect bandgap, with the CBM located between the *Γ* and *M*, and the VBM located between *K* and *Γ*. Similarly, as shown in Figure 3b, the monolayer SiS also contained the indirect bandgap, although both the CBM and VBM were located between *K* and *Γ*. As indicated by the bandgap values of Blue P (2.77 eV) and SiS (3.00 eV), lights with wavelengths shorter than 448 nm and 413 nm, respectively, could be absorbed by these two materials, respectively. A heterojunction formed by monolayer Blue P and monolayer SiS would maintain the band structural characteristics of single layers in the P-S or Si-P interface, and have narrower bandgaps than those in single-layer Blue P or SiS. In the Si-P interface, the CBM and VBM are located between *K* and *Γ* (Figure 3c), generating an indirect bandgap of 1.74 eV. In the P-S interface, the indirect bandgap is 2.30 eV, involving the CBM between *Γ* and *M* and the VBM between *K* and *Γ* (Figure 3d). Due to the bandgaps of the Si-P-2 and P-S-4 heterostructure, 1.74 eV and 2.30 eV, respectively, their absorption spectra are likely to be narrower than 713 nm and 539 nm, respectively. These absorption spectra are considerably expanded compared to those of monolayer Blue P and SiS which can only absorb UV light. In addition to UV light, visible light could also be harvested by the Blue P/SiS van der Waals heterostructures. Specifically, the Si-P interface with stacking order 2 (Si-P-2) could use almost the whole range of the visible light spectrum, suggesting high photocatalytic efficiency.

Unlike traditional 2D photocatalytic materials with symmetrical structures on both surfaces, an intrinsic dipole could be induced by the heterojunction within the bilayer material of Blue P and SiS, generating an internal electric field along the z-axis vertical to the surfaces of this photocatalyst. In this internal electric field, electrons and holes could be effectively separated. Additionally, the locations of energy bands on surfaces could be shifted via band bending, which is beneficial for satisfying the energy requirement for the redox reaction of water. Thus, this internal electric field improves the efficiency of hydrogen production from water splitting under light. The internal electric field (*E_ef_*) and the electrostatic potential difference (∆*Φ*) can be expressed by the following two equations, respectively, along with intrinsic dipole (***P***), dielectric constant (ε), surface area (*S*), and junction’s thickness (*d*) [12].

(1) $$\begin{array}{c} E_{ef}=\frac{P}{\varepsilon Sd}\; \end{array}$$

(2) $$\begin{array}{c} \begin{array}{c} ∆\Phi =\frac{eP}{\varepsilon S}\; \end{array} \end{array}$$

Equations (1) and (2) show linear positive correlations between ***P*** and *E_ef_*, as well as between ***P*** and ∆*Φ*, indicating that an increase in the intrinsic dipole ***P*** can increase *E_ef_* and ∆*Φ*. By combining the two equations above, Equation (3) indicates that a larger computed ∆*Φ* is associated with a larger internal electric field.

(3) $$\begin{array}{c} E_{ef}=\frac{∆\Phi }{ed}\; \end{array}$$

The value of ∆*Φ* can be obtained by the difference between the potentials on the $\left(001\right)$ and $\left(00\bar{1}\right)$ surface. For instance, there is a potential difference, 0.318 eV, between the two surfaces of monolayer SiS (Figure S3a,b). An intrinsic electric field is likely to exist, as indicated by the potential association between *E_ef_* and ∆*Φ* in Equation (3). The direction of *E_ef_* is from the $\left(001\right)$ surface for the reduction reaction toward the $\left(00\bar{1}\right)$ surface for the oxidation reaction. Additionally, monolayer Blue P does not vary in structure and potential on the two surfaces, indicating that no intrinsic electric field is involved in monolayer Blue P (Figure S3c,d). The electrical variation between SiS and Blue P is associated with their different atomic structures. Along the z-axis, monolayer Blue P has a symmetrical structure with only P atoms on both surfaces. However, monolayer SiS contains either Si atoms or S atoms on different surfaces, resulting in ∆*Φ* and *E_ef_* along the z-axis.

**Figure 4:**
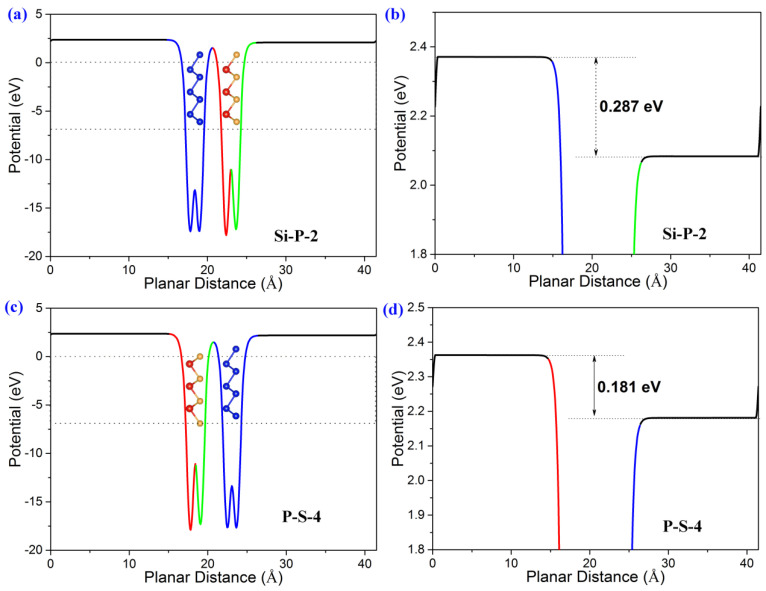
The planar average potential of the Blue P/SiS van der Waals heterostructures. (**a**,**b**) are the Si-P-2 stacking order. (**c**,**d**) are the P-S-4 stacking order. (**b**,**d**) are enlarged sections of (**a**,**c**).

Stacking asymmetrical monolayer SiS and symmetrical monolayer Blue P can establish a SiS/Blue P heterojunction with an asymmetrical structure along the z-axis, displaying ∆*Φ* between the two surfaces of the bilayer material (Figure 4). Given that the SiS/Blue P heterojunction is similar to monolayer SiS in ∆*Φ*, monolayer SiS is the main contributor to the electric field properties of this heterojunction. In addition, the intrinsic electric field values may vary depending on the interface, because the ∆*Φ* of the Si-P-2 stacking heterostructure is 0.287 eV, while the ∆*Φ* of the P-S-4 stacking heterostructure is 0.181 eV (Figure 4b,d). Moreover, the potential on the $\left(001\right)$ surface is lower than that on the $\left(00\bar{1}\right)$ surface, suggesting the existence of an intrinsic electric field from the $\left(001\right)$ to $\left(00\bar{1}\right)$ surface. 

For the photocatalyst used in water photolysis to generate hydrogen, the energy locations of bands are critical for evaluating photocatalytic efficiency. Materials with the appropriate locations of VBM and CBM are required for the electron-hole pair to drive redox reactions. Shen’s study proposed a mechanism of overall water splitting in two-dimensional polarized heterostructures [27]. Driven by the intrinsic electric field, photogenerated electrons would migrate to one surface of the heterostructures to participate in the hydrogen evolution reaction (HER), while photogenerated holes transfer to the opposite surface to facilitate the oxygen evolution reaction (OER). For a photocatalyst to efficiently achieve overall water splitting, its band edges must meet the thermodynamic requirements for water splitting redox potentials. Specifically, the VBM value should be less positive than the oxidation potential of water, while the CBM value should be more positive than the reduction potential of water. In a material with an intrinsic electric field, the VBM and CBM can be computed using Equations (4) and (5) [12], respectively, which involve the energy location of empty level *Φ* (∞), the energy location of the Fermi level (*E_F_*), and the energy of the bandgap (*E_gap_*).

(4) $$\begin{array}{c} \begin{array}{c} \begin{array}{c} E_{VBM}=\Phi \;\left(\infty \right)-E_{F} \end{array} \end{array} \end{array}$$

(5) $$\begin{array}{c} \begin{array}{c} \begin{array}{c} E_{CBM}=E_{VBM}+E_{gap} \end{array}\; \end{array} \end{array}$$

**Figure 5:**
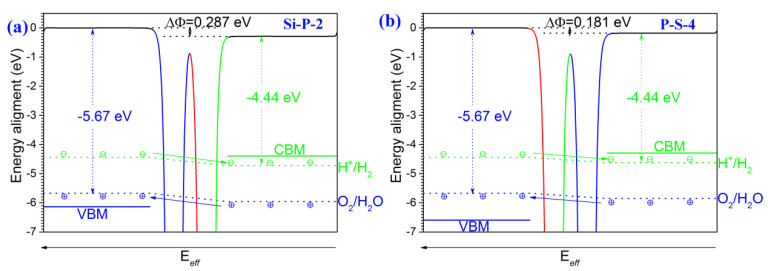
The band alignment of the Blue P/SiS van der Waals heterostructures. The (**a**) Si-P-2 stacking order. The (**b**) P-S-4 stacking order.

The energy levels for redox reactions would be bent by the intrinsic electric field, caused by the inequivalent electrostatic potentials on the $\left(001\right)$ and $\left(00\bar{1}\right)$ surface (Figure 5). Following the separation of electrons and holes under the field, in the Si-P-2 stacking heterostructure (Figure 5a), the VBM is located at −6.131 eV on the $\left(00\bar{1}\right)$ surface. This VBM value is less positive by 0.461 eV than the oxidation potential, allowing the reaction below to occur at the holes for O_2_ generation.

(6) $$\begin{array}{c} \begin{array}{c} 2H_{2}O+4h^{+}\to O_{2}+4H^{+} \end{array} \end{array}$$

Besides, the CBM is located at −4.391 eV on the $\left(001\right)$ surface, which is more positive by 0.336 eV than the reduction potential. Therefore, *H*_2_ production can occur through the reaction shown below. The variation between the VBM and oxidation potential is close to the variation between the CBM and reduction potential, suggesting that the system is in equilibrium.

(7) $$\begin{array}{c} 4H_{2}O+4e^{+}\to \;2H_{2}+4OH^{-} \end{array}$$

In the P-S-4 stacking heterostructure (Figure 5b), the CBM located at −4.291 eV is more positive by 0.330 eV than the reduction potential, while the VBM located at −6.594 eV is less positive by 0.924 eV than the oxidation potential. Therefore, the redox reaction of water can take place.

The ability for light absorption is one of the critical properties of photocatalysts. As the first step of photocatalysis, a high absorption rate across a wide range of the light spectrum by the ideal photocatalyst can generate sufficient photogenerated charge carriers. In the solar spectrum, the proportions of UV light, visible light and IR light are approximately 7%, 50% and 43%, respectively. Common photocatalysts usually only absorb UV light, so the solar utility rate is low. Expanding the absorption spectra of photocatalysts is one of the urgent issues to be solved.

The absorption coefficient *α* (*ω*) of monolayer Blue P, monolayer SiS and the Blue P/SiS heterojunction can be calculated using Equation (8), which involves the dielectric constants of the real part, $\varepsilon_{1}(\omega )$, and the imaginary part, $\varepsilon_{2}(\omega )$.

(8) $$\alpha \;\left(\omega \right)=\sqrt{2}\omega {\left(\sqrt{{{(\varepsilon }_{1}(\omega ))}^{2}+{{(\varepsilon }_{2}(\omega ))}^{2}}-\varepsilon_{1}\left(\omega \right)\right)}^{\frac{1}{2}}$$

**Figure 6:**
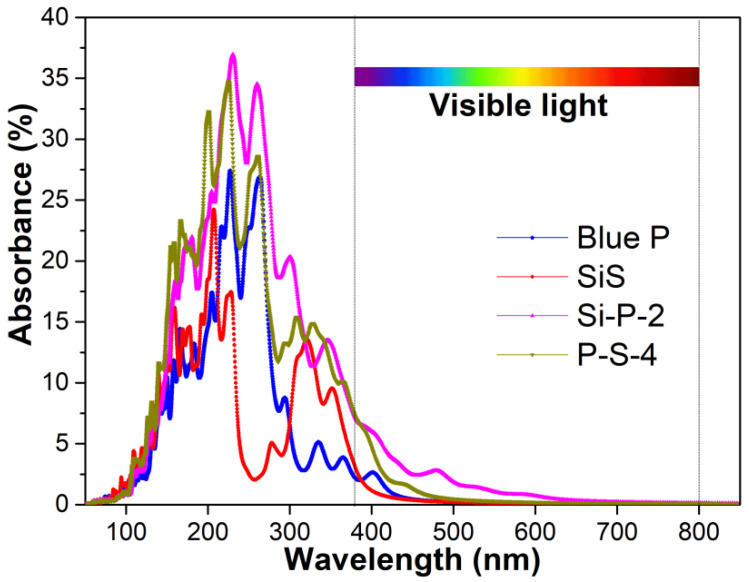
The absorbance of monolayer Blue P, monolayer SiS, and the Blue P/SiS van der Waals heterostructures with Si-P-2 and P-S-4 stacking orders.

The absorption spectra of monolayer Blue P and monolayer SiS are mainly in the UV range (Figure 6), since their bandgaps are 2.77 eV and 3.00 eV, respectively. Heterojunctions containing Si-P-2 or P-S-4 stacking can absorb light in a broader spectrum than monolayer Blue P and SiS, including UV light and part of the visible light spectrum. The absorption spectrum of the Si-P-2 stacking heterostructure with a narrow bandgap is the broadest among the evaluated materials in this work, allowing visible light to be harvested. Apart from the range of the absorption spectrum, the absorption values at the same wavelength are considerably promoted in the heterojunction with Si-P-2 (36.9%) and P-S-4 (34.7%), compared with monolayer SiS (24.2%) and Blue P (27.4%) (Figure 6). In summary, applying heterojunctions with Si-P-2 or P-S-4 stacking may benefit the efficiency of photocatalysts, due to their expanded absorption spectra and increased absorption values.

## 3. Method

The first principal calculation based on the density functional theory (DFT) was conducted in this work, using the Vienna ab initio simulation package (VASP) [69] and projector augmented wave (PAW) potentials [70]. Generalized gradient approximation (GGA) and the Perdew–Burke–Ernzerhof (PBE) functional were applied to describe the exchange correlation functionals [71]. The energy cutoff was 600 eV. During the structure optimization, the convergence thresholds for energy and force were set to be lower than 10^−6^ eV and 10^−3^ eV/Å, respectively. A vacuum region without atoms, which is larger than 25 Å along the z-axis, was set to prevent the periodic effects in the z-direction of the 2D material. The van der Waals interaction was described using the DFT-D3 method [72,73,74]. The stability of the 2D polarized Blue P/SiS heterostructure was studied by the ab initio molecular dynamics (AIMD) simulations [75]. The canonical ensemble method was adopted to set the temperature of 300 K. The total simulation time was 1000 fs, and the time interval was 1 fs. Blue P/SiS heterostructure with 5 × 5 × 1 supercells were formed. The K points meshing method in the first Brillouin zone employed the Gamma center method [76], with the K points mesh density set to 12 × 12 × 1 during structure optimization, and 15 × 15 × 1 for calculating the stable state. The band structures were calculated using the Heyd–Scuseria-Ernzerhof (HSE06) method [77] because this method is more accurate than the PBE method. The dipole moment was corrected in calculating the planer average potential. Additionally, VASPKIT [78] software was used to analyze the optical absorption spectrum, while VESTA software was used to visualize the atomic structure.

## 4. Conclusions

In conclusion, 2D polarized Blue P/SiS van der Waals heterostructures were formed based on monolayer Blue P and polar monolayer SiS with different stacking interfaces, including the Si-P and P-S interface. The Si-P-2 or P-S-4 stacking order contributes to the most stable heterostructure with the Si-P or P-S interface. The bandgaps of the Si-P-2 and P-S-4 heterostructures are 1.74 eV and 2.30 eV, resulting in their expanded absorption spectra. The electrostatic potential differences of the Si-P-2 and P-S-4 stacking heterostructures are 0.287 eV and 0.181 eV, respectively. An intrinsic electric field is established with the direction from the $\left(001\right)$ to the $\left(00\bar{1}\right)$ surface, which helps separate photo-generated electron–hole pairs. The band alignments of Blue P/SiS heterostructures are suitable for water splitting because the VBM values are less positive than the oxidation potential of water, while the CBM values are more positive than the reduction potential of water. Therefore, the results of this study suggest that 2D polarized Blue P/SiS heterostructures could be a potential novel photocatalyst for hydrogen generation by water splitting under a wide UV and visible light spectrum.

## Figures and Tables

**Table 1:** The lattice parameter (*a*), bond length of P-P (*d_P-P_*), Si-S (*d_Si-S_*), total thickness (*d_T_*), interlayer distance (*d_I_*), bandgap (*E_g_*), and HSE06 bandgap (*E_g_^HSE06^*).

Structure	*a*	*d_P-P_*	*d_Si-S_*	*d_T_*	*d_I_*	*E_g_*	*E_g_^HSE06^*	Ref.
	(Å)	(Å)	(Å)	(Å)	(Å)	(eV)	(eV)	
Blue P	3.27	2.26		1.24		1.95	2.77	This work
Blue P	3.27	2.26					2.78	[66]
Blue P	3.27	2.26				1.97	2.74	[67]
SiS	3.30		2.32	1.33		2.20	3.00	This work
SiS	3.31		2.33			2.19	3.03	[68]
SiS	3.30		2.32	1.33		2.20	3.00	[50]
Si-P-2 heterostructure	3.29	2.27	2.32	5.91	3.35	1.07	1.74	This work
P-S-4 heterostructure	3.29	2.26	2.32	5.91	3.35	1.52	2.30	This work

## Data Availability

The data that support the findings of this study are available from the corresponding author upon reasonable request.

## Supplementary Materials

The following supporting information can be downloaded at: https://www.mdpi.com/article/10.3390/molecules29184355/s1, Figure S1. The various stacking orders of Blue P/SiS van der Waals heterostructures. (a) Si-P-1, (b) Si-P-2, (c) Si-P-3, (d) Si-P-4, (e) Si-P-5, (f) Si-P-6, (g) P-S-1, (h) P-S-2, (i) P-S-3, (j) P-S-4, (k) P-S-5 and (l) P-S-6; Figure S2. The energy of the Blue P/SiS van der Waals heterostructures of (a) P-S and (b) Si-P interface various stacking orders; Figure S3. The planar average potential of (a,b) monolayer SiS, (c,d) monolayer Blue P. (b) and (d) are enlarged section of (a) and (c).
