# Supplementary material for: Two-Dimensional Polarized Blue P/SiS Heterostructures as Promising Photocatalysts for Water Splitting
Source: Molecules. 2024 Sep 13;29(18):4355. doi: 10.3390/molecules29184355 (PMC11434051; doi:10.3390/molecules29184355)
Supplement: Supplementary file 1 [file molecules-29-04355-s001.zip › molecules-3155534-supplementary.pdf]

Supporting Information

Two dimensional polarized Blue P/SiS heterostructures as promising photocatalyst for water splitting

Yin Liu[1], Di Gu[1,2*], Xiaoma Tao[3], Yifang Ouyang[3], Chunyan Duan[2*],Guangxing Liang[4]

1. Department of Physics, School of Science, Guangdong University of Petrochemical Technology, Maoming, Guangdong 525000, People's Republic of China.
2. School of New Energy and Environmental Protection Engineering, Foshan Polytechnic, Foshan 528137, People's Republic of China.
3. School of Physical Science and Technology, Guangxi University, Nanning 530004, People's Republic of China.
4. Shenzhen Key Laboratory of Advanced Thin Films and Applications, Key Laboratory of Optoelectronic Devices and Systems of Ministry of Education and Guangdong Province, State Key Laboratory of Radio Frequency Heterogeneous Integration, College of Physics and Optoelectronic Engineering, Shenzhen University, Shenzhen 518060, Republic of China.

* Correspondence: gudi@gdupt.edu.cn (D.G.); dcyan@fspt.edu.cn (C.D.)

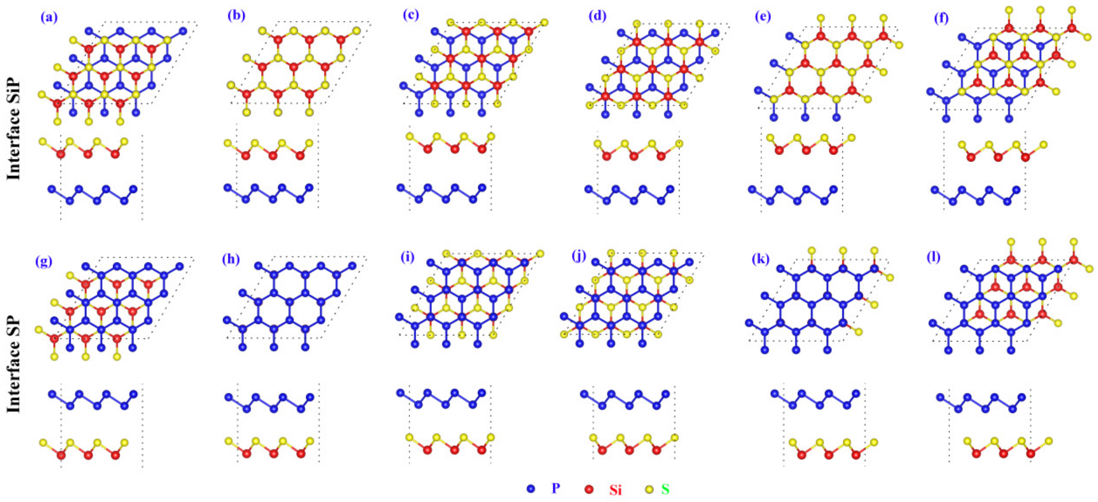

Figure S1. The various stacking orders of Blue P/SiS van der Waals heterostructures.

(a) Si-P-1, (b) Si-P-2, (c) Si-P-3, (d) Si-P-4, (e) Si-P-5, (f) Si-P-6, (g) P-S-1, (h) P-S-2, (i) P-S-3, (j) P-S-4, (k) P-S-5 and (l) P-S-6.

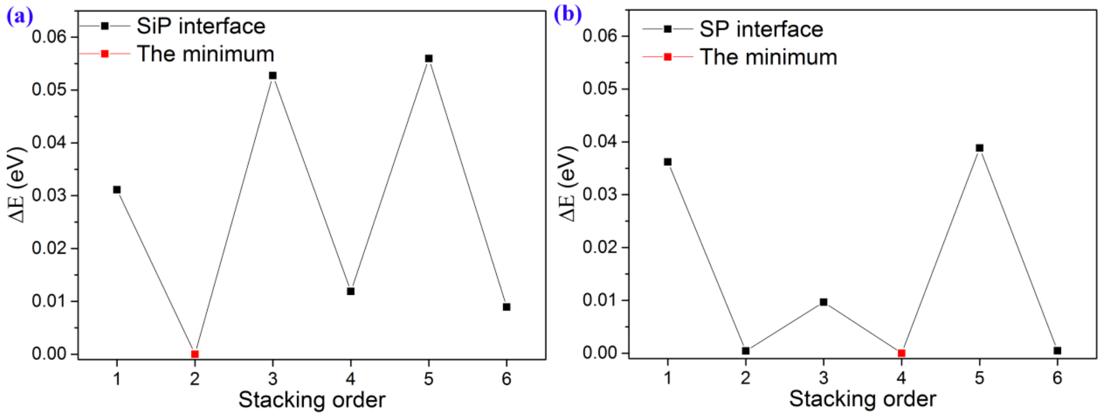

Figure S2. The energy of the Blue P/SiS van der Waals heterostructures of (a) P-S and (b) Si-P interface various stacking orders.

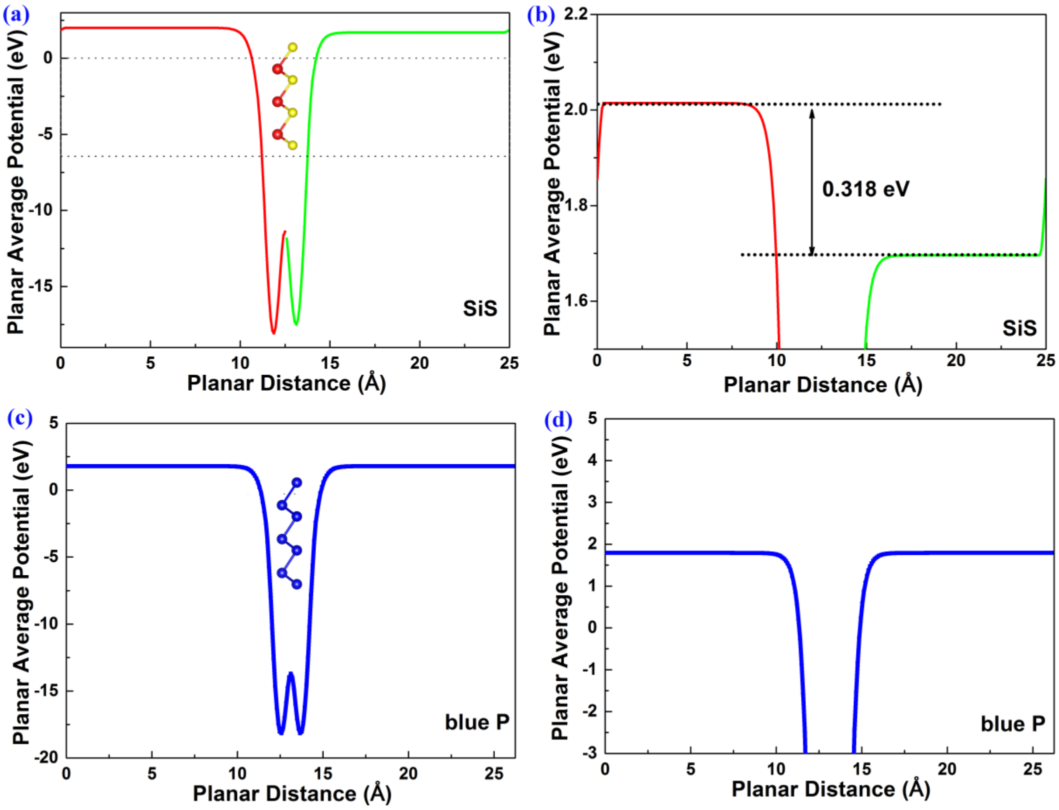

Figure S3. The planar average potential of (a, b) monolayer SiS, (c, d) monolayer Blue P. (b) and (d) are enlarged section of (a) and (c).
